# Induction and Recovery of the Viable but Nonculturable State of Hop-Resistance *Lactobacillus brevis*

**DOI:** 10.3389/fmicb.2018.02076

**Published:** 2018-10-15

**Authors:** Junyan Liu, Yang Deng, Thanapop Soteyome, Yanyan Li, Jianyu Su, Lin Li, Bing Li, Mark E. Shirtliff, Zhenbo Xu, Brian M. Peters

**Affiliations:** ^1^School of Food Science and Engineering, South China University of Technology, Guangzhou, China; ^2^Department of Clinical Pharmacy, College of Pharmacy, University of Tennessee Health Science Center, Memphis, TN, United States; ^3^College of Food Science and Engineering, Qingdao Agricultural University, Qingdao, China; ^4^Home Economics Technology, Rajamangala University of Technology Phra Nakhon, Bangkok, Thailand; ^5^Department of Cell Biology, Harvard Medical School, Boston, MA, United States; ^6^State Key Laboratory of Food Science and Technology, Jiangnan University, Wuxi, China; ^7^Guangdong Province Key Laboratory for Green Processing of Natural Products and Product Safety, Guangzhou, China; ^8^School of Chemical Engineering and Energy Technology, Dongguan University of Technology, Dongguan, China; ^9^Department of Microbial Pathogenesis, School of Dentistry, University of Maryland, Baltimore, MD, United States; ^10^Overseas Expertise Introduction Center for Discipline Innovation of Food Nutrition and Human Health (111 Center), Guangzhou, China

**Keywords:** *Lactobacillus brevis*, VBNC state, hop-resistance, beer spoilage, VBNC cells recovery

## Abstract

*Lactobacillus brevis* is a major hop-resistance bacterium which poses significant challenge for the brewing industry, mainly due to the difficulty or incapability in detection by routine culturing methodology and its beer spoilage ability.This study aimed at investigating the VBNC state of a hop-resistance strain, *L. brevis* BM-LB13908. The culturable, total and viable numbers of *L. brevis* cells were calculated by MRS agar plate counting, acridine orange direct count (AODC) method and Live/Dead BacLight bacterial viability kit with fluorescence microscope. VBNC formation was induced by 189 ± 5.7 days under low-temperature storage or 27 ± 1.2 subcultures by continuous passage in beer, and VBNC cells induced by both strategies were recovered by adding catalase. In addition, insignificant difference in beer-spoilage ability was found in 3 states of *L. brevis*, including logarithmic growing, VBNC and recovered cells. This is the first study on the formation of VBNC state for *L. brevis* and beer-spoilage ability of both VBNC and recovered cells, which indicate *L. brevis* strain could cause beer spoilage without being detected by routine methodologies. The results derived from this study may support further study on *L. brevis* and other hop-resistance bacteria, and guidance on beer spoilage prevention and control, such as improvement for brewers on the microbiological quality control by using the improved culture method with catalase supplementation.

## Introduction

As one of the most common beverage, beer has high microbiological stability and is considered to be safe, as it contains various microorganisms unable to survive in beer. Beer contains high ethanol (0.5 to 10% w/w) and carbon dioxide concentration (approximately 0.5% w/w), low pH (3.8–4.7), low concentrations of nutritive substances and extremely low oxygen content (<0.1 ppm), as well as hop bitter compounds (approximately 17–55 ppm of iso-α-acids) (Sakamoto and Konings, 2003; Suzuki et al., 2006). Hop components serve the desirable bitter flavor, characteristic aroma, and apply bacteriostatic effects on most Gram-positive bacteria (Simpson and Fernandez, 1992, 1994; Simpson, 1993). However, in spite of such unfavorable conditions for growth of microorganisms, a number of bacterial species (primarily Lactobacilli) are tolerant to hop compounds and capable of growing in hopped beer, thus designated as hop-resistance bacteria. Some Lactobacilli have been well studied and established to be one of the leading causes of turbidity and acidity in beer. In addition, formation of the viable but nonculturable (VBNC) state under extreme environments enables Lactobacilli strains to cause false-negative detection (Suzuki et al., 2006; Deng et al., 2015). VBNC cells are incapable of either growth on routine bacteriological culturing media or formation of normal colonies, but remain viable and retain metabolic activity (Oliver, 2005). If beer contaminated by VBNC strains has been delivered to commercial markets, even small numbers of hop-resistance bacteria will recover and eventually impart off-flavor and turbidity to beer. Up to date, five species of *Lactobacillus* strains have been verified for formation of VBNC state, including *L. lindneri* (Suzuki et al., 2006; Liu et al., 2017a), *L. casei* (Liu et al., 2017c), *L. plantarum* (Liu et al., 2017b), *L. paracollinocides* (Suzuki et al., 2006) and *L. acetotolerans* (Deng et al., 2015), which had been induced to enter into the VBNC state by beer subculture treatment or low-temperature storage.

As an important and most frequently discovered beer spoilage bacterium, *Lactobacillus brevis* is capable of producing end or by products of carbohydrate fermentation including lactic acid and acetic acid as significant influential factors on the flavors of beer, as well as biogenic amines which are associated with food intoxication, blood pressure increasing, migraines, dystonias, Parkinson’s disease, schizophrenia, drug addiction, mental disorders and neurodegenerative disease (Premont et al., 2001; Marcobal et al., 2006; Miao et al., 2017; Xu et al., 2017; Lin et al., 2018). *L. brevis* has been frequently identified within the beer sector as a spoilage bacterium (Garofalo et al., 2015), which is also physiologically versatile and responsible for various concerns in beer such as super-attenuation due to its fermentation of dextrins and starch (Lawrence, 1988). Importantly, *L. brevis* is commonly resistant to hop compounds, and the antibacterial effects of hop compounds and the mechanism(s) responsible for hop resistance have been previously studied in *L. brevis* strains (Suzuki et al., 2002).

This study aimed to investigate the induction, recovery and beer-spoilage capacity of VBNC state *L. brevis* strain BM-LB13908 which shows strong resistance to hop compounds.

## Materials and Methods

### Bacterial Strains and Growth Conditions

The present *L. brevis* strain (designated BM-LB13908) had been isolated from finished beer sample (pH 4.5, ethanol ≥ 3.6% v/v, bitterness units seven) and maintained as a glycerol stock stored at −80°C. Whole genome sequencing was performed for this strain and the draft genomic sequence was deposited in GenBank under accession number LTDY00000000. *L. brevis* BM-LB13908 shows strong hop resistance and contains the hop resistant gene *horA* in its genome. A small amount of stock was spread onto MRS agar (Oxoid, United Kingdom) and incubated at 26°C for 3 days to obtain isolated colonies. A single colony was transferred to 1 mL of MRS broth and incubated anaerobically at 26°C for 24 h prior to further experiments.

### Formation of VBNC State

Based on the environment beer-spoilage bacteria encountered during beer brewing (continuous passage) and storage (low-temperature), two strategies, including low-temperature storage and continuous passage in beers were selected to conduct the formation of VBNC state of strain *L. brevis* BM-LB13908. Low temperature or cold treatment have also been frequently reported to induce the VBNC formation of various bacteria in previous studies (Mary et al., 2002; Gupte et al., 2003; Kong et al., 2004, 2014; Sun et al., 2008; Dinu and Bach, 2011; Griffitt et al., 2011; Patrone et al., 2013; Zeng et al., 2013; Magajna and Schraft, 2015; Liu et al., 2017d). For the former, cells of *L. brevis* BM-LB13908 in logarithmic growth phase were harvested at 4°C (centrifugation at 5000 rpm for 15 min) and washed twice with phosphate buffer (PBS). Then the washed cells were filtered through a 0.22 μm Millipore membrane filter and resuspended in 10 mL of degassed beer at a final density of 10^7^ cells/mL. The bacterial cells were further maintained at 0°C for the formation of VBNC state.

Continuous passage in beer was performed as described previously (Deng et al., 2015), with slight modification. In brief, approximately 10^7^ cells of *L. brevis* BM-LB13908 in logarithmic growth phase were harvested at 4°C (centrifugation at 5000 rpm for 15 min) and washed twice with PBS. Then the washed cells which marked as 0^#^ subculture were resuspended by 10 mL of degassed beer at a final density of 10^7^ cells/mL and anaerobically cultured at 26°C. After 7 days incubation, the cells were marked as 1^#^ subculture and harvested, washed, and resuspended in 10 mL of fresh degassed beer. The process was repeated every 7 days.

### Cell Populations Quantification

In the current study, the definition of “VBNC state” is the incapability of microbial growth on MRS agar until 3 days of incubation at 26°C. For both induction processes, the culturable cell number was accessed by inoculating 100 μL of bacterial culture on MRS agar according to a conventional plate culture procedure (Suzuki et al., 2008). After 3 days of incubation at 26°C, cell culturability was determined by CFU counting.

Number of total cells was determined every 7 days by AODC method (Hobbie et al., 1977). In brief, bacterial cultures were centrifuged and washed twice with PBS. Then the washed cell samples were serially diluted in 0.5 M sodium PBS at pH 7.0, fixed with formalin (final concentration, 2% v/v), and stained with 0.01% w/v acridine orange. After 2 min incubation, the suspension was filtered onto a 0.22 μm Millipore membrane filter. The filter was then examined under an Olympus BH-2 fluorescence microscope (Olympus, Japan).

Cellular viability determination was further conducted by the Live/Dead BacLight bacterial viability kit (Molecular Probes, United States), followed by observation under fluorescent microscope (Berney et al., 2007). In brief, the fluorescent dyes propidium iodide (20 mM in DMSO) and SYTO 9 (3.34 mM in DMSO) were mixed in a 2:1 ratio. Washed cell samples (500 μL) were stained with 1.5 μL of dye mixture at room temperature in the dark for 20 min. Stained cell samples were then immediately filtered onto a 0.22 μm Millipore membrane filter. The filters were placed on glass slides with 10 μL mounting oil (Millipore) followed by sealing of the coverslips. Slides were immediately taken for viewing under an Olympus BH-2 fluorescence microscope (Olympus, Japan). Cellular culturability and viability measurement were performed once a week.

### Recovery of the VBNC Cells

After the VBNC state induction process, chemicals addition was performed for recovery of VBNC *L. brevis* cells. Oxidative stress was encountered by the VBNC *L. brevis* cells under low temperature and harsh beer condition (Chattopadhyay et al., 2011), adding catalase was applied to relief the oxidative stress and promote the recovery of VBNC cells (Jallouli et al., 2010; Kong et al., 2014). Tween and vitamins, which have been reported to successfully recover VBNC bacteria, especially beer spoilage bacteria (Ramaiah et al., 2002; Sakamoto and Konings, 2003), were also used. One hundred microliters of *L. brevis* VBNC cells induced by either strategies were plated onto MRS agar supplemented with Tween-20, Tween-80, vitamin C, vitamin B2, and catalase (Sigma-Aldrich, United States) at final concentrations of 0.1%, 0.1%, 5 g/L, 5 g/L, and 800 U/plate. Simultaneously, MRS agar with no supplements and addition of heat (60°C for 15 min) denatured catalase were controls. After incubation at 26°C, daily examination for the microbial growth and colony formation on agar plate was performed, with the day of first observation of colonies recorded as detection time.

### Beer-Spoilage Capacity Evaluation

The “growth in beer test” had been performed to study the beer-spoilage capability of *L. brevis* BM-LB13908 (Sakamoto and Konings, 2003). Approximately 10^5^ cells/mL of logarithmic growing, VBNC, and recovered cells were inoculated into finished degassed beer under sterile conditions at 26°C. Non-inoculated beer was served as control. The inoculated and non-inoculated beers were examined visually every day for turbidity for 30 days. At day 30, lactic acid and acetic acid concentrations were detected by reversed-phase high performance liquid chromatography (HPLC) and quantified by external standard method (Deng et al., 2015). Fifty μL of cell samples collected by centrifugation and filter was injected into the 1100 series HPLC system (Agilent, United States). Compounds were separated on a 250 × 4.60 mm, 4 μm Synergi Hydro-RP 80 Å column (Phenomenex, Lane Cove, Australia) at 30°C. The mobile phase was 0.43% orthophosphoric acid with a flow rate of 1.0 mL/min, and elution was monitored by 210 nm UV detection.

### Statistical Analysis

All the experiments were conducted in triplicate. Data are presented as mean ± standard deviation (SD) of three independent biological replicates. Statistical comparisons were made by one-way analysis of variance followed by Tukey’s comparison test (XLstat software). The *p*-value < 0.05 was considered to be significant in this study.

## Results and Discussion

### Formation of VBNC State

**FIGURE 1:**
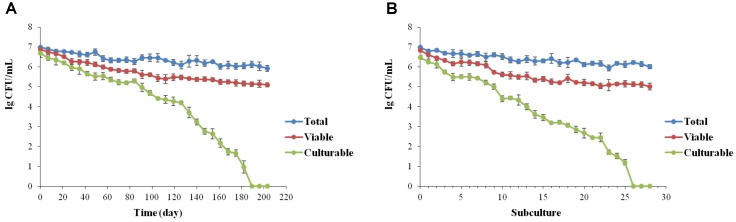
Induction of the VBNC state of *Lactobacillus brevis* BM-LB13908 by low-temperature storage (0°C) **(A)** or continuous passage in beer **(B)**, respectively.

For low-temperature storage, despite a low decrease in viable cells showing a final concentration of 10^5^ cells/mL, whole population of *L. brevis* cells were no longer culturable after 189 ± 5.7 days (**Figure 1A**). According to the continuous passage in beers, formation of VBNC state of *L. brevis* strain BM-LB13908 was found with 27 ± 1.2 subcultures (**Figure 1B**). The present results had verified the induction on VBNC state of *L. brevis* by simulating conditions during beer brewing. It was also found that the culturability of *L. brevis* declined significantly during conditions induced by beer subculture or low temperature, indicating the VBNC *L. brevis* could be potentially neglected in brewery environments due to incapability of detection by traditional culture methods. As *Lactobacillus* strains were concerned, up to date, a total of five species have been validated for VBNC state formation, including *L. lindneri* (Suzuki et al., 2006; Liu et al., 2017a), *L. casei* (Liu et al., 2017c), *L. plantarum* (Liu et al., 2017b), *L. paracollinocides*, (Suzuki et al., 2006) and *L. acetotolerans* (Deng et al., 2015), which had been induced to enter into VBNC state by either low-temperature storage or beer subculture treatment. Consequently, this is the first the formation and recovery of VBNC state by the species of *L. brevis* as the most common beer spoilage and hop resistance strain.

### VBNC Cells Recovery

Viable but nonculturable cells of *L. brevis* strain BM-LB13908 induced by low-temperature storage or beer subculture had been treated by addition of Tween-20 or-80, vitamin C or B2, with <10 CFU/mL culturable cells acquired. However, 16 colonies (160 CFU/mL cells) appeared on MRS agar supplemented with catalase at day 3. Thus, VBNC *L. brevis* cells regained culturability on media containing catalase. Importantly, no colony appeared on the MRS agar plates with no supplementation and addition of heat-denatured catalase. Heat denaturation of catalase deferred the recovery process, verifying catalase addition is capable of recovery of VBNC *L. brevis* cells. Such finding suggested the potential improvement for brewers on the microbiological quality control by culture methodology with catalase supplementation. It has been reported that transfer of cells to nutrient-rich environment initiates a metabolic imbalance, thus leading to a rapid production of superoxide and free radicals (Bloomfield et al., 1998). Furthermore, the antibacterial function of hop compounds has been reported to associate with efficient redox reactivity and cause cellular oxidative damage (Behr and Vogel, 2010). One possible explanation is the VBNC *L. brevis* cells are under environmental stress and sensitive to detoxify superoxide during active phenotypic adaptation to beer environment with high concentration of hop bitter compounds, and antioxidant capacity of catalase may alleviate these stress conditions, leading to the recovery of VBNC cells by addition of catalase.

### Beer-Spoilage Capacity

According to the results, the logarithmic, VBNC, and recovered cells of *L. brevis* strain BM-LB13908 were capable of causing turbidity in beer within a length of approximate 7 days (data not shown), suggesting its maintenance of beer-spoilage capability in both VBNC and recovered state. No turbidity was observed in the non-inoculated beer. In addition, higher level of both lactic acid and acetic acid were found after incubation for 30 days, which may eventually lead to beer acidizing (**Table 1**). It indicates both VBNC and recovered cells retained as similar level of viability and metabolic activity as logarithmic cells, which was in accordance with previous studies (Rahman et al., 2001; Jolivet-Gougeon et al., 2006; Du et al., 2007; Imazaki and Nakaho, 2009; Ordax et al., 2009; Buck and Oliver, 2010; Patrone et al., 2013). As the most commonly identified spoilage microorganism in beer industry, *L. brevis* is capable of VBNC formation under common food storage condition (low-temperature), recovery by addition of catalase, as well as maintained production of lactic acid and acetic acid, which may be the leading cause for a wide range of beer spoilage cases. In addition, it indicated that the normal, VBNC, and recovered *L. brevis* cells are capable of causing spoilage within the storage life of beer.

**Table 1:** Results of beer-spoilage ability test.

Strain	State	Turbidity	Lactic acid (mg/L)	Acetic acid (mg/L)
Fresh beer		−	86.45 ± 23.61^a^	75.77 ± 25.23^a^
*Lactobacillus brevis* BM-LB13908	Normal	+	191.86 ± 38.15^b^	189.57 ± 41.75^b^
	VBNC	+	181.28 ± 37.87^b^	172.39 ± 35.92^b^
	Recovered	+	201.45 ± 32.46^b^	162.29 ± 31.83^b^

*^+^, positive; −, negative. The different superscript letters in the same column indicate difference among concentrations at 0.05 level.*

## Conclusion

In conclusion, this is the first report on the VBNC induction and recovery of *L. brevis*, as well as beer-spoilage ability of both VBNC and recovered cells. For *L. brevis* strain BM-LB13908 in the present study, VBNC formation and recovery, as well as maintenance of beer spoilage capability have been verified, which indicated *L. brevis* strain could cause beer spoilage due to incapability of detection by routine methodologies. The results derived from this study may aid in further study on *L. brevis* and other hop-resistance bacteria, and guidance on the prevention and control of beer spoilage, such as improvement for brewers on the microbiological quality control by using the improved culture method with catalase supplementation.

## Author Contributions

ZX and YD conceived of the study and participated in its design and coordination. JL and YD performed the experimental work. LL, JS, and BL analyzed the data. JL, YL, BP, MS, and ZX prepared and revised this manuscript. All authors reviewed and approved the final manuscript.

## Conflict of Interest Statement

The authors declare that the research was conducted in the absence of any commercial or financial relationships that could be construed as a potential conflict of interest. The reviewer MC and handling Editor declared their shared affiliation.
